# The chromatin remodeler ALC1 underlies resistance to PARP inhibitor treatment

**DOI:** 10.1126/sciadv.abb8626

**Published:** 2020-12-18

**Authors:** Szilvia Juhász, Rebecca Smith, Tamás Schauer, Dóra Spekhardt, Hasan Mamar, Siham Zentout, Catherine Chapuis, Sébastien Huet, Gyula Timinszky

**Affiliations:** 1 MTA SZBK Lendület DNA Damage and Nuclear Dynamics Research Group, Institute of Genetics, Biological Research Centre, 6276 Szeged, Hungary.; 2 Univ Rennes, CNRS, IGDR (Institut de Génétique et Développement de Rennes), UMR 6290, BIOSIT, UMS 3480, F-35000 Rennes, France.; 3 Biomedical Center, Bioinformatics Unit, Ludwig Maximilian University of Munich, 82152 Planegg-Martinsried, Germany.; 4 Institut Universitaire de France, Paris France.

## Abstract

Poly(ADP-ribose) polymerase (PARP) inhibitors are used in the treatment of BRCA-deficient cancers, with treatments currently extending toward other homologous recombination defective tumors. In a genome-wide CRISPR knockout screen with olaparib, we identify ALC1 (Amplified in Liver Cancer 1)—a cancer-relevant poly(ADP-ribose)-regulated chromatin remodeling enzyme—as a key modulator of sensitivity to PARP inhibitor. We found that ALC1 can remove inactive PARP1 indirectly through binding to PARylated chromatin. Consequently, ALC1 deficiency enhances trapping of inhibited PARP1, which then impairs the binding of both nonhomologous end-joining and homologous recombination repair factors to DNA lesions. We also establish that ALC1 overexpression, a common feature in multiple tumor types, reduces the sensitivity of BRCA-deficient cells to PARP inhibitors. Together, we conclude that ALC1-dependent PARP1 mobilization is a key step underlying PARP inhibitor resistance.

## Teaser

ALC1 mobilizes trapped PARP1, allowing the subsequent recruitment of downstream repair factors to initiate efficient repair.

## INTRODUCTION

Poly(ADP-ribose) polymerase 1 (PARP1) is a DNA damage sensor important for maintaining genomic integrity. PARP1 recognizes and binds both single-strand breaks (SSBs) and double-strand breaks (DSBs), triggering its ADP-ribose polymerase activity (*1*). Upon DNA damage, PARP1 poly(ADP-ribosyl)ates (PARylates) several DNA damage repair–associated proteins and chromatin components that are crucial for efficient DNA damage repair. As expected, the loss of PARP1 or the inhibition of PARP activity sensitizes cells to DNA-damaging agents (*2*–*5*).

PARP inhibitors (PARPis) were shown to be particularly toxic for cells deficient in homologous recombination (HR) repair factors BRCA1 (Breast cancer type 1 susceptibility protein) and BRCA2 (Breast cancer type 2 susceptibility protein) even in the absence of exogenous DNA damage, a phenomenon with great therapeutic potential because of the high prevalence of BRCA deficiency in tumor cells (*6*–*8*). The observation that the synthetic lethality between BRCA deficiency and PARPi treatment is abrogated by the loss of PARP1 revealed that the inhibited DNA-bound PARP1 is the toxic product—the phenomenon called PARP trapping—rather than deficient DNA damage signaling in the absence of PARylation (*4*).

Olaparib was the first PARPi to be approved for the treatment of BRCA-deficient breast and ovarian cancers (*9*, *10*). The therapeutic use of PARPis brought increased interest in elucidating genetic alterations that lead to sensitivity or resistance to PARP inhibition. The loss of several HR and interstrand cross-link repair components leads to PARPi sensitivity, signifying that HR is essential for the faithful repair of the increased level of DNA lesions induced by PARP1 trapping (*7*, *8*, *11*). In HR-deficient cells, these lesions are handled by the error-prone nonhomologous end-joining (NHEJ) pathway that ultimately leads to chromosome aberrations and cell death. The synthetic lethality between PARPis and BRCA1 deficiency can be reversed by the loss of the NHEJ factor 53BP1 (Tumor Protein P53 Binding Protein 1), which reactivates HR in a PALB2 (Partner And Localizer Of BRCA2)–dependent manner (*12*). Recently, it has become apparent that PARPi sensitivity can be attributed to malfunction of pathways other than defective HR. For example, defective ribonucleotide excision repair due to the loss of ribonuclease H2 activity was recently identified as a source of DNA lesions that can cause PARP1 trapping (*13*). Moreover, loss of PARylation factors such as histone PARylation factor 1 (HPF1) and poly(ADP-ribose) glycohydrolase (PARG) can sensitize cells or promote resistance to PARPi, respectively (*14*, *15*). In the current study, we used a genome-wide CRISPR knockout screen with olaparib to identify other molecular mechanisms that could modulate sensitivity to PARPi treatment to which the therapeutic spectrum could potentially be extended.

## RESULTS

### CRISPR-based knockout screen identifies ALC1 deficiency as a source PARPi sensitivity

**Fig. 1:**
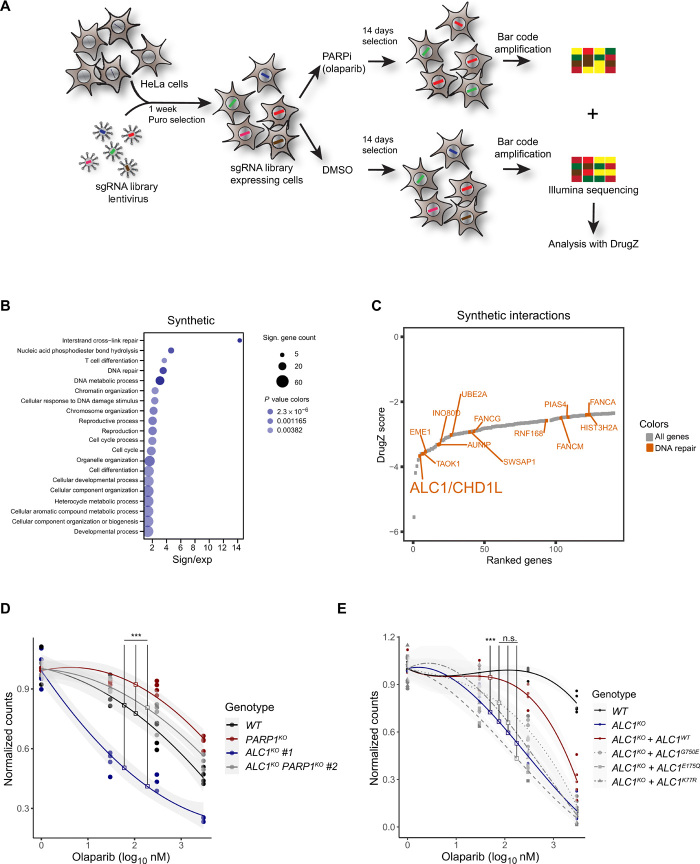
A genome-wide CRISPR knockout screen reveals ALC1 as a gene conveying PARP inhibitor resistance. (**A**) Schematic of the CRISPR screen. (**B**) Dot plot showing the enrichment of 20 Gene Ontology processes. The size of the dots represents the number of significant genes associated with the Gene Ontology term, and the color of the dots represents the *P* value. (**C**) Scatterplot of DrugZ analysis result of the genes showing synthetic interactions with olaparib. Genes annotated with functions in DNA repair are colored red. (**D**) Clonogenic cell survival assay of U2OS WT, *ALC1^KO^*, *PARP1^KO^*, and *ALC1^KO^ PARP1^KO^* double knockout cells after a 24-hour treatment with olaparib. (**E**) Clonogenic cell survival assay of U2OS WT and *ALC1^KO^* expressing mCherry-ALC1 variants after a 24-hour treatment with olaparib. Graphs in (D) and (E) include all data points (*n* = 3 to 5) and fitted curves with 95% confidence intervals (gray shading). Asterisks indicate *P* values obtained by polynomial regression (n.s., not significant; ***P < 0.001). Model summary is provided in table S2.

To identify previously unknown factors that modulate cell sensitivity to the clinically approved PARPi, olaparib, we infected wild-type (WT) HeLa cells with the GeCKOv2 whole-genome CRISPR-based knockout pooled library targeting each gene with six single-guide RNAs (sgRNAs) (*16*, *17*). Knockout cells were subjected to olaparib at a concentration yielding approximately 40% survival, enriching the proportion of cells resistant to the drug treatment while depleting knockout cells with increased sensitivity to treatment. Genomic DNA was collected after 14 days of treatment, and sgRNA cassettes were amplified and deep-sequenced. Sequence output from dimethyl sulfoxide (DMSO) control and olaparib-treated samples were analyzed using the DrugZ algorithm (*18*) to identify candidate genes influencing sensitivity to olaparib (Fig. 1, A to C; fig. S1, A and B; and table S1). Examination of Gene Ontology terms of genes whose loss led to PARPi resistance revealed a number of cell cycle– and mTOR (mammalian target of rapamycin)–associated processes. Deficiency in PARP1 or PARG also conveyed resistance to PARPi (fig. S1, A and B), in agreement with previous reports (*4*, *15*). Conversely, we found that loss of several genes belonging to DNA repair processes, in particular cross-link repair, was associated with increased sensitivity to PARPi including previously identified genes involved in HR such as FANCM (Fanconi anemia complementation group M) (fig. 1, B and C) (*11*). BRCA1 and BRCA2, whose loss was previously shown to provide PARPi sensitivity (*7*, *8*), failed to score in our screen due to reduced cell fitness upon loss of each of these two genes (fig. S1C and table S1). One of the strongest candidate genes whose loss increased sensitivity to PARPi was the poly(ADP-ribose) (PAR)–dependent chromatin remodeler ALC1 (Amplified in Liver Cancer 1)/CHD1L (Chromodomain Helicase DNA Binding Protein 1 Like)..

ALC1 is a member of the SNF2 superfamily of chromatin remodelers. It is unique among the hits for synthetic lethality as a protein that directly binds PAR, the product of PARP1 activity, promoting its activation (*19*, *20*). ALC1 is amplified in many solid tumors and is associated with tumor progression (*21*). Previous studies demonstrated the correlation between ALC1 overexpression and poor patient survival in non–small cell lung cancer (*22*) as well as patient chemotherapy resistance in human hepatocellular carcinoma (*23*). Studies based on DT40 model cell line investigated the role of ALC1 in base excision repair with PARP1 cooperation and showed that both *PARP1^KO^* and *ALC1^KO^*, *PARP1^KO^* double knockout cells had similarly impaired SSB repair. Moreover, cells expressing ATPase-dead ALC1 displayed hypersensitivity to various DNA-damaging agents (*24*), while the depletion of ALC1 increased cell sensitivity to various DNA-damaging agents [H_2_O_2_, ultraviolet (UV), and phleomycin] (*19*, *25*). To validate the impact of ALC1 loss on PARPi sensitivity, which was also found in another screen (*13*), we generated *ALC1^KO^* in U2OS cell lines using CRISPR-Cas9–based gene editing and studied clonogenic cell survival in the presence of varying olaparib concentrations. All three independent *ALC1^KO^* cell lines tested showed sensitivity to olaparib even at low concentrations, confirming their synthetic lethality with PARPi (Fig. 1D and fig. S1D). To address whether the synthetic lethality between *ALC1^KO^* and PARPi required the PARP1 protein, we generated *ALC1^KO^ PARP1^KO^* double knockout cell lines. *PARP1^KO^* showed resistance to olaparib treatment in agreement with previous reports, and we also observed that the loss of PARP1 in *ALC1^KO^* cells decreased sensitivity to PARPi (Fig. 1D and fig. S1D). These results are consistent with PARPi toxicity requiring the presence of PARP1 (*4*). This observation was also confirmed in *ALC1^KO^* cells by RNA interference (RNAi)–mediated down-regulation of PARP1 (fig. S1E). Furthermore, *ALC1^KO^* cells showed sensitivity to the PARPis veliparib and niraparib, which have lower and higher trapping potential, respectively, as compared to olaparib (fig. S1, F and G) (*26*). The differences in inhibitor concentrations at which the hypersensitivity of the *ALC1^KO^* cells can be detected mirror the relative PARP1 trapping potential of the three inhibitors (*27*). Last, the sensitivity of *ALC1^KO^* cells to PARPi treatment could be partially rescued when complemented with ALC1-WT (Fig. 1E and fig. S1H) but not with a PAR-binding mutant of ALC1 that cannot recruit to sites of DNA damage (fig. S1I) or with ALC1 ATPase-deficient mutants that recruit to sites of DNA damage as efficiently as ALC1-WT (fig. S1I) but are unable to remodel the chromatin (*5*). Together, these results identify ALC1 as a crucial component providing resistance to PARPi. Failure of cells to form Rad51 foci has been used as a predictor of PARPi sensitivity. HR-deficient cells such as BRCA1 and BRCA2 null cells do not form foci irrespective of PARPi treatment, while their WT counterparts show robust Rad51 foci formation in the presence of PARPi (*7*, *8*, *28*). We saw an increase in Rad51 foci formation in WT cells treated with PARPi as well as increased Rad51 foci levels in *PARP1^KO^* cells irrespective of PARPi treatment, as previously described (fig. S1, J and K) (*29*). In *ALC1^KO^* cells, we observed elevated levels of Rad51 foci compared to WT cells in the absence of PARPi and that this did not increase upon the addition of PARPi (fig. S1, J and K). This result indicates that PARPi sensitivity of *ALC1^KO^* cells may differ from HR deficiency, a common cause of PARPi sensitivity.

The observation that a PAR-responsive enzyme is important for cell survival in the presence of PARPi where PARylation is blocked seems counterintuitive. Nevertheless, we observed that olaparib concentrations that were toxic for *ALC1^KO^* cells did not fully suppress ALC1 recruitment to DNA lesions in WT cells likely due to residual PARP1 catalytic activity (fig. S1, L and M). This incomplete inhibition of PARylation signaling upon PARPi treatment may also explain why the deletion of PARG causes PARPi resistance, while PARG degrades PAR, the formation of which should be blocked by PARPis (*15*).

### PARP inhibition induces DNA damage and cell cycle arrest in ALC1 knockouts

In the absence of genomic stress, most nuclear PARylation is limited to S phase where unligated Okazaki fragments provide the trigger for PARP1 binding and activation (*30*). In addition to this role in DNA replication, PARP1 activation has been reported to be important for the regulation of HR components and replication fork reinitiation after replication-induced fork stalling and subsequent DNA damage (*31*, *32*). Conversely, PARP inhibition was reported to induce abnormal acceleration of replication fork elongation, leading to DNA damage accumulation (*33*). Together, these data prompted us to assess the impact of ALC1 and PARP inhibition in S-phase progression.

To assess replication fork dynamics, we looked for colocalization of EdU (5-ethynyl-2′-deoxyuridine) and IdU (5-iodo-2′-deoxyuridine) after an initial pulse labeling of active replication foci using the nucleotide analog EdU and a second pulse labeling of ongoing replication with IdU (fig. S2A). While we were able to see DNA damage–induced replication fork arrest after high-dose x-ray [8 gray (Gy)] treatment, shown as a lack of IdU incorporation after EdU pulse labeling (fig. S2B), we found that *ALC1^KO^*, *PARP1^KO^*, and *ALC1^KO^ PARP1^KO^* double knockout cells showed IdU incorporation at EdU signals after olaparib treatment, indicating that PARP inhibition does not impair S-phase progression in any of the tested cell lines (fig. S2C).

**Fig. 2:**
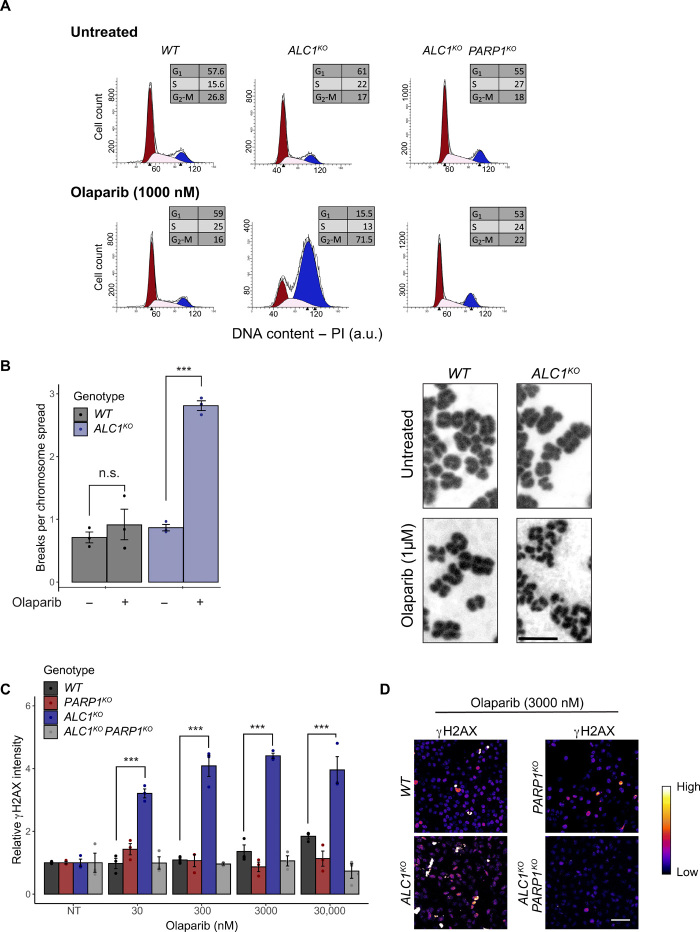
PARP1 inhibition induces chromosome aberrations and cell cycle arrest in *ALC1^KO^* cells. (**A**) Representative flow cytometry profiles of cells with the indicated genotypes with or without 1 μM olaparib treatment for 24 hours. The distribution of cells in G_1_, S, or G_2_-M is indicated in the inserted boxes. PI, propidium iodide; a.u., arbitrary units. (**B**) Cells were grown for 24 hours in the presence or absence of 1 μM olaparib. After 24 hours, cells were treated with colchicine to arrest the cells in M phase and collected after 6 hours. Left: Chromosome aberrations were counted in 40 chromosome spreads per sample. Right: Representative images of chromosome spreads of WT and *ALC1^KO^* cells with or without olaparib treatment. Scale bar, 2 μm. (**C**) Relative γH2AX intensity in *ALC1^KO^* and/or *PARP1^KO^* U2OS cells. The intensity of γH2AX signal was measured in untreated (NT) or olaparib-treated cells after 12 hours. (**D**) Representative images of the level of γH2AX in *ALC1^KO^* and/or *PARP1^KO^* U2OS cells after olaparib treatment. Scale bar, 30 μm. Graphs in (B) and (C) include all data points and mean ± SEM (*n* = 3). Asterisks indicate *P* values obtained by linear regression (***P < 0.001). Models in (C) were fitted independently for each concentration. Model summary is provided in table S2.

Next, we analyzed the *ALC1^KO^* cells by flow cytometry to further characterize the consequence of PARPi treatment on cell cycle progression. We find that a 24-hour olaparib treatment leads to the accumulation of *ALC1^KO^* cells arrested in the G_2_-M phase. In contrast, the cell cycle distribution of the olaparib-resistant *ALC1^KO^ PARP1^KO^* double knockouts was not affected by PARPi treatment similar to WT cells (Fig. 2A). In addition, in *ALC1^KO^* cells, PARPi treatment resulted in an increase of both chromosome breaks, as shown by chromosome spread (Fig. 2B and fig. S2D), and DNA breaks as measured by alkaline comet tail moment length (fig. S2E). Consistent with this increase in DNA lesions, *ALC1^KO^* cells treated with PARPi also displayed an increase in the phosphorylated fraction of histone H2AX indicative of elevated DNA damage signaling (Fig. 2, C and D). These accumulating DNA lesions are feasibly the cause of the cell cycle arrest at G_2_-M, ultimately leading to the synthetic lethality observed between PARPi treatment and ALC1 deficiency.

### Sensitivity of ALC1-deficient cells to genomic stress is increased in the presence of PARPis

**Fig. 3:**
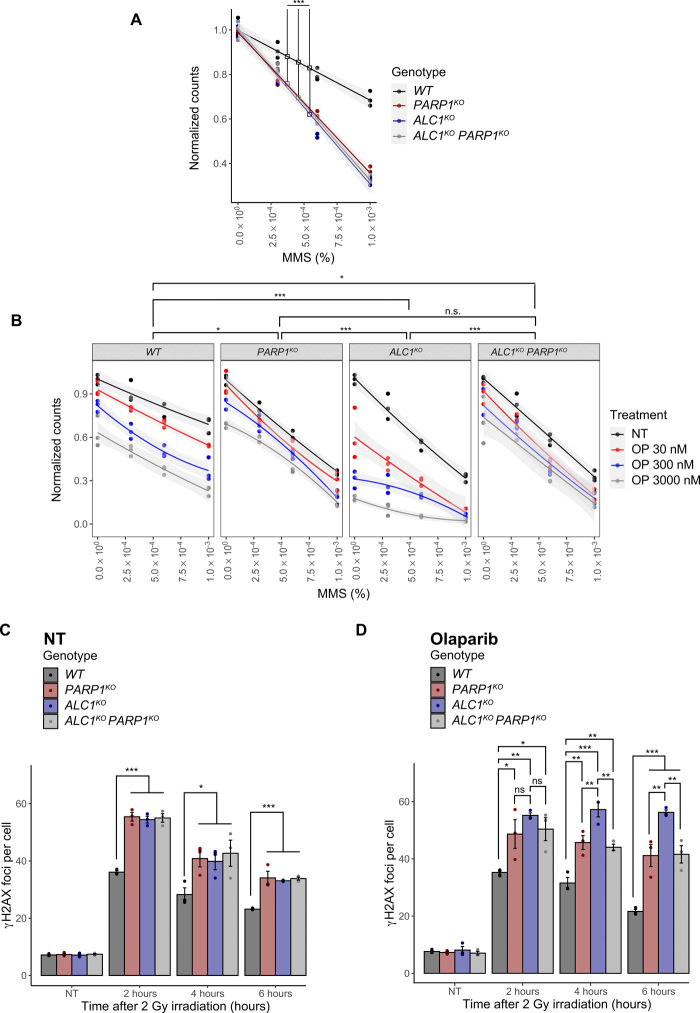
The hypersensitivity of ALC1-deficient cells to DNA-damaging agents is enhanced by PARP1 inhibition. (**A**) Clonogenic cell survival assay of *ALC1^KO^* and/or *PARP1^KO^* U2OS cells. DNA damage was induced by MMS for 1 hour. (**B**) Clonogenic cell survival assay of *ALC1^KO^* and/or *PARP1^KO^* U2OS cells. PARP1 inhibition was induced by olaparib (OP) treatment for 24 hours, and then DNA damage was induced by MMS for 1 hour. NT, non-treated. (**C** and **D**) Quantification of γH2AX foci in *ALC1^KO^* and/or *PARP1^KO^* U2OS cells. Where indicated, cells were treated with 1 μM olaparib for 1 hour before DNA damage induction with x-ray irradiation (2 Gy). γH2AX foci were counted at different time points after irradiation. NT, non-treated. Graphs in (A) and (B) include all data points (*n* = 3) and fitted curves with 95% confidence intervals (gray shading). Asterisks indicate *P* values obtained by linear or polynomial regression, respectively (*P < 0.05, **P < 0.01, and ***P < 0.001). *P* values in (B) correspond to three-way interaction terms comparing genotypes. Graphs in (C) and (D) include all data points and mean ± SEM (*n* = 3). Asterisks indicate *P* values obtained by linear regression fitted independently for each time point. Statistical summary is provided in table S2.

In a context of active PARylation signaling, both PARP1 and ALC1 are important for efficient DNA repair because cells lacking PARP1 or ALC1 both display similar hypersensitivity to various DNA damage stress (Fig. 3A and fig. S3, A to C). Noteworthy, knocking down or knocking out PARP1 in ALC1-deficient cells did not increase sensitivity to genotoxic stress, suggesting that PARP1 function in DNA repair occurs mainly via ALC1 (Fig. 3A and fig. S3, A to C). These findings are in contrast to cell sensitivity obtained in the presence of PARPi. *ALC1^KO^* cells show marked hypersensitivity to olaparib and were further sensitized by methyl methanesulfonate (MMS) treatment. WT, *PARP1^KO^*, and *ALC1^KO^ PARP1^KO^* double knockout cells have similar sensitivity to MMS in response to olaparib (Fig. 3B).

We also examined γH2AX foci formation after x-ray irradiation as a measure of DNA damage repair efficacy. In the absence of PARPi treatment, *PARP1^KO^*, *ALC1^KO^*, and *ALC1^KO^ PARP1^KO^* double knockout cells all showed similarly elevated γH2AX foci formed at each time point examined after x-ray irradiation, consistent with comparable defective repair in these knockout lines (Fig. 3C). Treatment with olaparib resulted in an elevated number of γH2AX foci in *ALC1^KO^* cells for prolonged periods of time after x-ray irradiation as compared to WT, *PARP1^KO^* and *ALC1^KO^ PARP1^KO^* double knockout cells (Fig. 3D). Consistent with the increase in γH2AX signal, *ALC1^KO^* treated with PARPi also displayed increased comet tail moment length (fig. S3D). These different results—showing that the acute repair defects displayed by *ALC1^KO^* cells in the presence of PARPi require the presence of PARP1—suggest a role of ALC1 in the regulation of PARP1 trapping at DNA lesions, which was shown to be the major source of PARPi-dependent cytotoxicity (*4*).

### ALC1 deficiency increases olaparib-induced PARP1 trapping

To gain insight into the molecular mechanisms underlying the ALC1-dependent modulation of PARPi sensitivity, we analyzed the characteristics of PARP1 binding to chromatin. First, we quantified the fraction of chromatin-bound mCherry-PARP1 after detergent preextraction in the absence of exogenous damage. In comparison to controls, cells depleted of ALC1 displayed an elevated fraction of chromatin-bound PARP1 upon olaparib treatment (fig. S4A). Similar behavior was observed upon depletion of BRCA1, which also displays PARP-dependent hypersensitivity to PARPi treatment (fig. S4A).

**Fig. 4:**
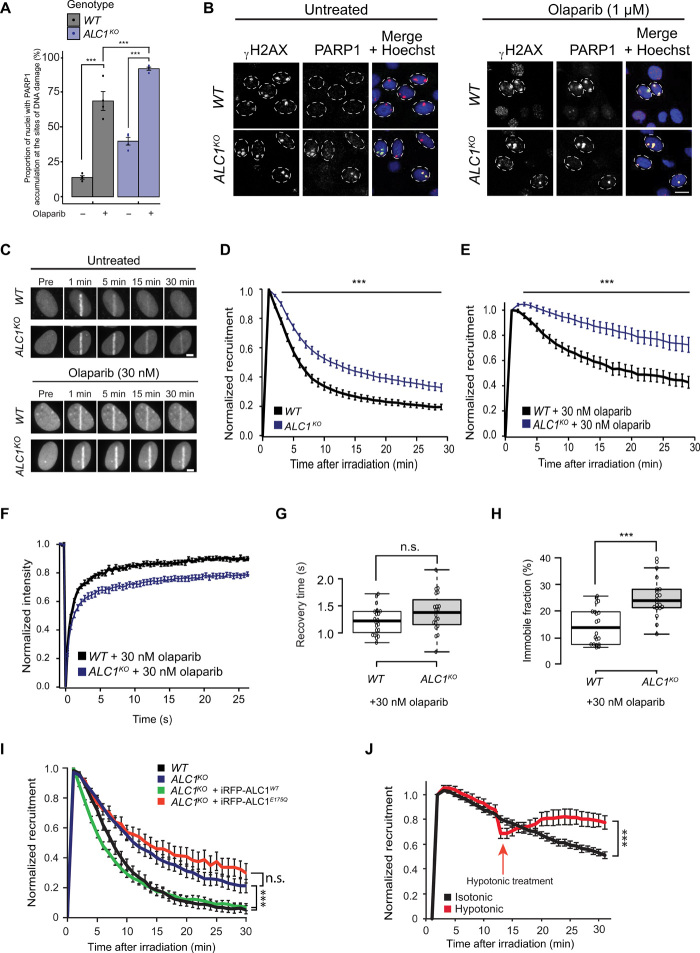
ALC1 deficiency increases olaparib-induced PARP1 trapping. (**A**) PARP1 at UV-induced DNA damage sites was quantified in WT and *ALC1^KO^* cells treated or not with 1 μM olaparib. (**B**) Representative images from (A). Scale bar, 10 μm. (**C**) Representative images showing GFP-PARP1 accumulation at sites of laser-induced DNA damage in WT and *ALC1^KO^* cells treated or not with 30 nM olaparib. Scale bar, 5 μm. (**D** and **E**) Quantified accumulation of GFP-PARP1 at DNA damage in WT or *ALC1^KO^* cells untreated (D) or treated with 30 nM olaparib (E). (**F**) Normalized FRAP curves of GFP-PARP1 at sites of DNA damage 30 min after irradiation in WT and *ALC1^KO^* cells. (**G**) Recovery time and (**H**) the immobile fraction of GFP-PARP1 at the break were calculated from the FRAP curves. (**I**) Quantified accumulation of GFP-PARP1 E988K at DNA damage in U2OS WT or *ALC1^KO^* cells expressing iRFP670-ALC1 variants. (**J**) Quantified accumulation of mCherry-PARP1 at DNA damage in *ALC1^KO^* cells ± hypotonic treatment. Graphs include all data points and mean ± SEM. Asterisks in (A) indicate *P* values obtained by linear regression (***P < 0.001). Model summary is provided in table S2. *P* values for (D) to (J) were obtained using an unpaired Student’s *t* test with Bonferroni correction (***P < 0.001).

To assess whether the increase in chromatin-bound PARP1 in *ALC1^KO^* cells is related to PARP1 trapping at DNA lesions, we measured the fraction of endogenous PARP1 bound to sites of DNA damage 30 min after UV micropore irradiation (Fig. 4, A and B) or to sites of x-ray–induced DNA damage (fig. S4B). While, as expected, PARP inhibition increased PARP1 trapped at DNA lesions, we found more PARP1 trapped in *ALC1^KO^* cells as compared to WT controls regardless of the presence of PARPis (Fig. 4, A and B, and fig. S4B). We measured similar trapped PARP1 fraction in the BRCA2-deficient cell line after olaparib treatment upon DNA damage induction (fig. S4C).

Next, to test whether these differences in PARP trapping are consecutive to a role of ALC1 in the mobilization of PARP1 from the sites of DNA damage, we examined in living cells the kinetics of accumulation to sites of laser microirradiation of GFP-PARP1, whose efficient recruitment to damage relies on intact DNA binding domain [fig. S4D and (*34*)]. In the presence of active PAR signaling, the progressive release of PARP1 from DNA lesions was slower in *ALC1^KO^* compared to control cells (Fig. 4, C and D). Upon olaparib treatment, PARP1 release was markedly slowed in WT cells, and this release was again slower in ALC1-deficient cells (Fig. 4, C and E). In addition, we analyzed the dynamics of PARP1 turnover at sites of laser microirradiation in PARPi-treated cells by fluorescence recovery after photobleaching (FRAP) (Fig. 4F). The recovery curves revealed the presence of two populations of PARP1 molecules: a fast-exchanging population characterized by a recovery time of about 1 s and a more stably bound fraction that can be considered as immobile within the analyzed time frame. While the recovery time of the mobile PARP1 population was comparable in WT and *ALC1^KO^* cells (Fig. 4G), the immobile fraction, which likely represents trapped PARP1 molecules, was higher in ALC1-deficient cells (Fig. 4H). In addition, we measured the levels of PAR at sites of laser microirradiation by following the recruitment of the PAR-binding macrodomain of macroH2A1.1 and found that, while the peak PARylation levels in WT and *ALC1^KO^* cells are similar, PAR levels remain elevated for a longer time in *ALC1^KO^* cells. Moreover, *ALC1^KO^* cells show slightly elevated levels of PARylation in the presence of PARPi. Both situations are consistent with a slower release of PARylated PARP1 from sites of DNA lesions (fig. S4E).

ALC1 is activated when it binds PAR with its macrodomain (*19*, *35*, *36*), these PAR chains being present on PARP1 itself but also on histones located nearby the damage sites (*37*, *38*). Consequently, the contribution of ALC1 to PARP1 mobilization could be due to either a remodeling of the autoPARylated PARP1 proteins by the ALC1 remodelers that are directly bound it or a more distal impact of the activity of ALC1 molecules recruited along the chromatin fiber. To investigate these two hypotheses, we analyzed the mobilization of the PARP1-E988K mutant, which is only capable of producing mono–ADP-ribose on which ALC1 is unable to bind (*36*), from DNA lesions in WT and *ALC1^KO^* cells. To make such experiment conclusive, we first had to test whether PARP1-E988K could be PARylated in trans by WT PARP1, which could provide a binding site for ALC1. We used the PAR3H assay we previously developed (*39*) to assess the PARylated PARP1 binding to the LacI-anchored macrodomain of macroH2A1.1 enriched at a genome-integrated LacO-array. While WT PARP1 released from the DNA lesions readily enriches at the anchored macrodomain upon DNA damage (fig. S4, F and G), the E988K mutant PARP1, which shows dynamic turnover at the break (fig. S4H), does not accumulate at the anchored macrodomain, indicating that PARP1 E988K is not PARylated in trans by WT PARP1. Consequently, our observation that the PARP1 E988K mutant is mobilized slower in the absence of ALC1 (Fig. 4I) reveals that ALC1 can participate in the removal of PARP1 without directly binding to it. Moreover, this mobilization of PARP1 E988K requires the ATPase activity of ALC1 because the expression of WT ALC1, but not that of an ATPase-deficient mutant, in *ALC1^KO^* cells efficiently mobilized PARP1 E988K from the lesions.

One hypothesis that could explain how ALC1 indirectly mobilizes non-PARylated PARP1 is that the rapid chromatin relaxation induced by ALC1 activity at DNA lesions (*5*) facilitates PARP1 release. To investigate this possibility, we analyzed the impact on PARP1 mobilization of rescuing the defect in chromatin relaxation observed in the absence of ALC1 by bathing the cells with hypotonic medium, which is known to induce global chromatin opening (*40*), after damage induction. However, relaxing chromatin by hypotonic treatment did not accelerate PARP1 release but instead increased the amount of PARP1 accumulating at the break sites (Fig. 4J). This result, which is consistent with our previous observation that chromatin relaxation facilitates the binding of DNA binding proteins to DNA lesions (*41*), makes our initial hypothesis regarding the contribution of chromatin relaxation to PARP1 release unlikely and, thus, rather call for an alternative model in which PARP1 is “peeled off” from DNA through nucleosome sliding by ALC1 anchored on PARylated nucleosomes. Together, our results suggest that ALC1 indirectly promotes the removal of even inactive, non-PARylated PARP1 from DNA lesions through anchoring to PARylated chromatin, to avoid deleterious consequences associated with PARP trapping.

### In the absence of ALC1, neither HR nor NHEJ is able to efficiently resolve PARP1-DNA adducts

**Fig. 5:**
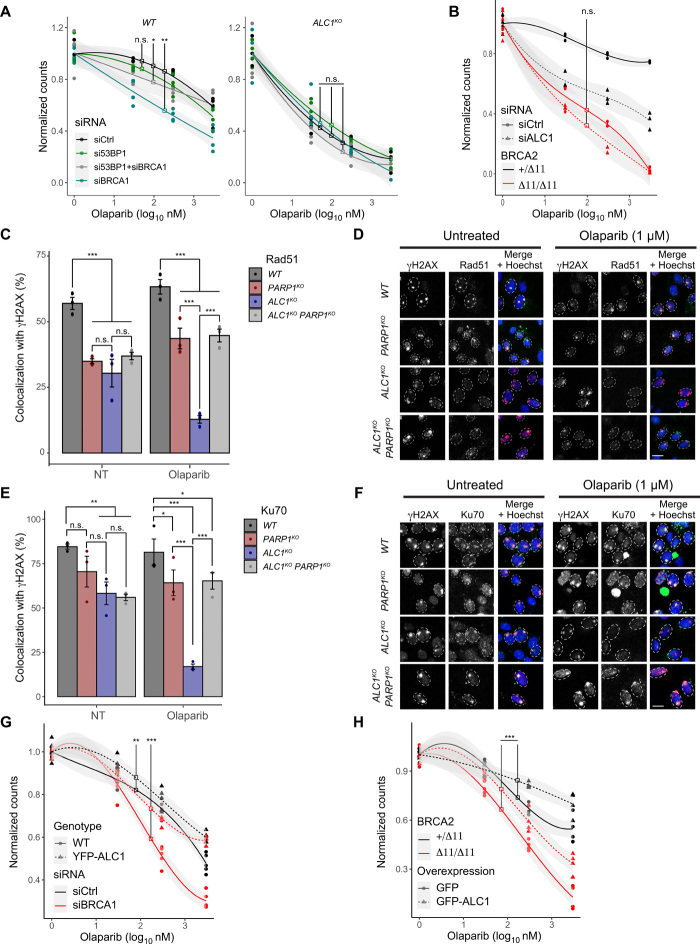
ALC1 acts upstream of DSB repair pathway choice. (**A**) Clonogenic cell survival assay of WT and *ALC1^KO^* U2OS cells transfected with siCtrl or siBRCA1 and/or si53BP1 treated or not with olaparib for 24 hours. (**B**) Clonogenic cell survival assay of DLD1-BRCA2^+/Δ11^ (BRCA2^+/−^) and DLD1-BRCA2^Δ11/Δ11^ (BRCA2^−/−^) cells transfected with siCtrl or siALC1 and treated or not with olaparib for 24 hours. (**C** to **F**) Quantification and representative images of Rad51 or Ku70 localization to UV-induced DNA damage sites in WT, *PARP1^KO^*, *ALC1^KO^*, and *PARP1^KO^ ALC1^KO^* double knockout cells, treated or not with olaparib (1 μM). Scale bar, 10 μm. (**G**) Clonogenic cell survival assay of WT and YFP-ALC1 overexpressing U2OS cells transfected with siCtrl or siBRCA1 and treated or not with olaparib for 24 hours. (**H**) Clonogenic cell survival assay of DLD1-BRCA2^+/Δ11^ (BRCA2^+/−^) and DLD1-BRCA2^Δ11/Δ11^ (BRCA2^−/−^) cells transfected with GFP-ALC1 and treated or not with olaparib for 24 hours. Graphs in (A), (B), (G), and (H) include all data points (*n* = 3) and fitted curves with 95% confidence intervals (gray shading). Graphs in (C) and (E) include all data points and mean ± SEM (*n* = 3). Asterisks indicate *P* values obtained by polynomial (A, B, G, and H) or linear regression (C and E) (*P < 0.05, **P < 0.01, and ***P < 0.001). Model summary is provided in table S2.

Next, we sought to determine how PARP trapping in *ALC1^KO^* cells affected DNA repair pathways. In many cases, sensitivity to PARPi arises from an imbalanced DSB pathway choice. In HR-deficient cells, PARPi toxicity is due to the activation of the error-prone NHEJ pathway for resolving PARP1-DNA adducts (*42*). Conversely, PARPi resistance upon loss of 53BP1 in BRCA1-deficient cells is the consequence of the reactivation of HR, which is able to faithfully repair the DNA lesions associated with PARP1-trapping (*12*). To gain more insight into the mechanism of the ALC1-synthetic lethal phenotype, we compared the impact of RNAi-mediated BRCA1 and 53BP1 depletion on olaparib treatment in WT and *ALC1^KO^* cells. In line with previous reports, olaparib is toxic in BRCA1-depleted WT cells, and the codepletion of 53BP1 and BRCA1 rescues the synthetic lethality with olaparib treatment (*43*). In contrast, the synthetic lethal phenotype seen in *ALC1^KO^* cells upon PARP1 inhibition remained unchanged after down-regulation of BRCA1 and/or 53BP1 (Fig. 5A and fig. S5A). In addition, we found that ALC1 depletion does not increase the sensitivity of cells suffering from BRCA2 deficiency to olaparib treatment (Fig 5B and fig. S5B). These observations suggest that ALC1 has to handle the PARP1-DNA adducts before the DSB repair pathway choice to allow the resolution of these lesions by either HR or NHEJ.

To more directly assess the impact of ALC1 on HR and NHEJ, we used two previously described reporter systems (fig. S5, C and D) (*44*). First, I-SceI–induced breaks repaired by HR in pGC-HeLa cells will restore a GFP cassette, allowing GFP fluorescence to act as a readout for HR efficiency (fig. S5C). Second, in the pEJ-HeLa reporter assay, I-SceI–induced breaks repaired by NHEJ will allow expression of a GFP cassette, providing a measure for NHEJ efficiency (fig. S5D). In agreement with previous reports, we verified that down-regulation of the HR factor BRCA1 decreased while down-regulation of the NHEJ factor Ku70 increased HR efficiency in the pGC-HeLa cells and that they had the opposite effect in the pEJ-HeLa reporter line (fig. S5, E to G). The down-regulation of ALC1 resulted in reduced efficiency of HR and NHEJ; however, the latter was not statistically significant, albeit in the absence of PARPi (fig. S5, E to G).

To study the effect of ALC1 deficiency in combination with PARPi on HR and NHEJ, we studied the recruitment of Ku70, an actor of NHEJ, as well as Mre11, Rad51, and phosphorylated replication protein A (pRPA), which are specific to HR, to sites of DNA damage induced by micropore UV irradiation. In the absence of PARPi, Rad51, Ku70, Mre11, and pRPA showed reduced accumulation to the γH2AX-labeled DNA damage sites in both *ALC1^KO^* and *PARP1^KO^* as well as the double knockout cells as compared to WT cells (Fig. 5, C to F, and fig. S5, H to K). Upon olaparib treatment, the accumulation of Rad51, Ku70, pRPA, and Mre11 was reduced even further at the sites of DNA damage in *ALC1^KO^* as compared to other cell lines, including the *ALC1^KO^ PARP1^KO^* double knockout cells. Together, these results show that ALC1 is important for mobilizing PARP1 and that, in its absence, trapped inhibited PARP1 interferes with the binding of downstream HR and NHEJ repair factors to the sites of DNA damage.

### ALC1 overexpression reduces the PARPi sensitivity of BRCA-deficient cells

ALC1 is an oncogene frequently overexpressed in cancer correlating with poor prognosis for patient survival (*21*–*23*). While the loss of ALC1 sensitizes cells to DNA-damaging agents and PARPi treatment (Figs. 1D and 3 and fig. S1, F and G), we aimed to address whether ALC1 overexpression would have the opposite effect. We found that ALC1 overexpression reduced sensitivity to various DNA-damaging agents (fig. S5, L to N) in agreement with previous studies (*22*, *23*) and increased the frequency of both HR and NHEJ in the reporter cell lines (fig. S5, O and P). Last, overexpression of ALC1 reduced the sensitivity to olaparib of WT and BRCA-deficient cells (Fig. 5, G to H, and fig. S5, Q and R). Notably, BRCA-deficient cells overexpressing ALC1 are almost as sensitive as WT cells to olaparib. This suggests that an increase of ALC1 expression in BRCA-deficient tumors would tend to reduce or even suppress the therapeutic window in which PARPi could be used to efficiently kill BRCA-deficient tumor cells while sparing their healthy counterpart.

## DISCUSSION

Synthetic lethal interactions between PARPis and BRCA-deficient tumors have been a focus for cancer therapies for a number of years (*6*–*8*, *45*, *46*). In addition to specific mutations in the BRCA1/2 genes, this therapeutic strategy has been extended to tumors showing defects in the HR pathway that phenocopy the loss of BRCA, a phenotype often referred to as BRCAness (*47*). In the current study, we identify the PAR-dependent chromatin remodeler ALC1 as a key modulator of sensitivity to the PARPi olaparib and show that loss of ALC1 impairs both the HR and NHEJ repair pathways. Furthermore, the hypersensitivity to PARPi treatment observed in *ALC1^KO^* cells is not rescued by down-regulating 53BP1, in contrast to what is reported for BRCA-deficient cells (*43*). These findings show that ALC1 deficiency does not display the classical BRCAness signature and that the sensitivity of *ALC1^KO^* cells to PARPi arises from defects at the very early stage of the DNA damage response, before the pathway choice between HR and NHEJ.

**Fig. 6:**
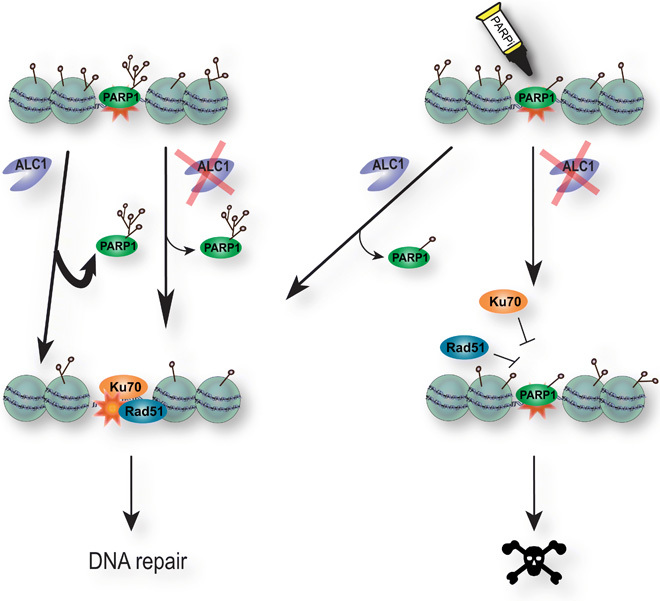
A model for the role of ALC1 in olaparib-mediated synthetic lethality. PARP1 is recruited to sites of DNA damage where it PARylates proteins in and around the break site, including itself. PARP1 removal from sites of damage involves a combination of autoPARylation and ALC1-dependent mobilization, which allows the recruitment of essential subsequent repair actors such as Ku70 or Rad51. While the impairment of either of the two modes of PARP1 mobilization does not have major deleterious consequences, inhibiting autoPARylation in ALC1-deficient cells fully blocks PARP1 release from DNA lesions, thus preventing the recruitment of downstream repair factors and ultimately leading to cell death.

Our results demonstrate that the chromatin remodeler ALC1 is involved in the timely mobilization of PARP1 from the DNA lesions. In the context of active PARylation signaling, the loss of ALC1 already affects PARP1 removal from the lesions, but alternative mechanisms such as PARP1 autoPARylation are sufficient to compensate for this defect, allowing the cell to proceed to DNA repair. In contrast, as soon as PARylation is impaired, ALC1 becomes essential for the mobilization of PARP1 from the sites of damage. Failure to achieve efficient PARP1 removal interferes with downstream steps in the DNA repair pathway, ultimately leading to cell death (Fig. 6). Our results reveal that the processivity of ALC1 along the chromatin fiber is able to peel PARP1 off the sites of damage. It should be noted that HPF1 deficiency—which abolishes the PARylation of histones—also leads to PARPi sensitivity as well as increased PARP1 trapping (*14*, *38*, *48*), which is consistent with ALC1 acting indirectly on PARP1 mobility through chromatin PARylation and subsequent nucleosome sliding.

ALC1 overexpression is a common trait of multiple tumors, often associated with poor prognosis (*23*). According to our findings, the risks are high that such tumors would display low responsiveness to PARPis, thus strongly arguing for a systematic analysis of the ALC1 expression level before the use of PARPi-driven cancer therapies. Noteworthy, the region 1q21 within chromosome 1, where the *ALC1* gene is located, is found amplified in many cancers including breast tumors (*49*) and is associated with resistance to chemotherapy treatment in ovarian cancers (*50*). Moreover, ALC1 was found overexpressed in ovarian carcinoma metastasis (*51*), a feature that is associated with shorter patient survival. This, together with our results, suggests that, in addition to a potential role as a predictive biomarker, there is also a call for the development of new therapeutic agents against ALC1. A first compound targeting this remodeler and showing some therapeutic potential against colorectal cancer has been developed very recently (*52*). On the basis of our data, this new agent should synergize the cytotoxic potential of the currently available PARPis.

## MATERIALS AND METHODS

### Cell lines and cell culture

All cells used here were cultured in Dulbecco’s modified Eagle’s medium (DMEM; Sigma-Aldrich) or RPMI supplemented with 10% fetal bovine serum (FBS), penicillin (100 μg/ml), streptomycin (100 U/ml), and 1% nonessential amino acid and maintained at 37°C in a 5% CO_2_ incubator unless otherwise stated. DLD1-BRCA2^+/Δ11^ (BRCA2^+/−^), DLD1-BRCA2^Δ11/Δ11^ (BRCA2^−/−^) (*53*), U2OS YFP-ALC1, U2OS *PARP1^KO^*, and U2OS *ALC1^KO^* #1 were generated previously (*5*). Additional U2OS *ALC1^KO^* cells were generated using CRISPR-Cas9 technology as previously described (*5*, *54*) using WT U2OS cells as the parental cell line. U2OS *ALC1^KO^ PARP1^KO^* double knockout cells were generated by knocking out ALC1 in *PARP1^KO^* U2OS cells. The sgRNA sequences used for targeting ALC1 (table S3) were designed using an online tool (*55*). HeLa PARP1-mCherry stable cells were generated by transfecting HeLa cells with PARP1-mCherry (*56*) and growing them in culture media supplemented with Geneticin (500 μg/ml) for 2 weeks. HeLa pGC and HeLa pEJ reporter cells were a gift from W. Mansour (*44*) and were cultured in DMEM with 10% FBS, penicillin (100 μg/ml), streptomycin (100 U/ml), puromycin (600 μg/ml), and G418 sulfate (800 μg/ml).

### RNAi and plasmid transfection

pYFP-macrodomain of macroH2A1.1 (*56*); pLacI-GFP trap, piRFP670-ALC1, and piRFP670-ALC1 E175Q (*39*); pPARP1-GFP, pPARP1-R34E-GFP, and pPARP1-R138E-GFP (*34*); pmEGFP-ALC1 and pmCherry-ALC1 G750E (*36*); and pmCherry-ALC1, pmCherry-ALC1 K77R, pmCherry-ALC1 E175Q, photoactivatable GFP (PAGFP)–H2B, and photoactivatable TagRFP (PATagRFP)–H2B (*5*) were previously described. PARP1 complementary DNA was amplified from PARP1-mCherry and PARP1 E988K-mcherry (primers in table S3) (*56*) and ligated into pmEGFP-C1, pDendra2-C1, or pmCherry-C1 between Bgl II and Xma I. Cells were transfected with plasmids using X-tremeGENE HP (Roche) or Xfect (Takara Bio) according to the manufacturer’s instructions.

Transfection of cell lines with specific small interfering RNAs (siRNAs) (table S4) was carried out using DharmaFECT (Dharmacon) transfection reagent according to the manufacturer’s instructions. Experiments were performed 48 hours after siRNA transfection. The down-regulation of the indicated genes was verified by Western blot using specific antibodies, which are detailed in table S5.

### Genome-wide CRISPR screen

A CRISPR-Cas9 genome-wide knockout screen was performed using the GeCKOv2 system as previously described (*16*, *17*). GeCKOv2 human CRISPR knockout library was amplified as described using New England Biolabs 5-alpha Electrocompetent *Escherichia coli.* For lentiviral production, 293T cells were transfected with amplified GeCKOv2 plasmid DNA, psPAX2 (a gift from D. Trono; Addgene, plasmid #12260), and pLP-eco env, a mouse Lentivirus envelope packaging vector (a gift from G. Schotta) using Xfect transfection reagent (Takara) according to the manufacturer’s instructions. Supernatant was collected 48 hours after transfection, filtered through a 0.45-μm Steriflip filter unit, and stored at −80°C. Virus titer [multiplicity of infection (MOI)] was calculated as previously described (*16*).

For the screen, HeLa cells stably expressing mCAT1 (mouse High affinity cationic amino acid transporter 1) (*57*) were transduced with the GeCKOv2 lentiviral library at an MOI of 0.3 and selected with puromycin (0.3 μg/ml) for 7 days as previously described (*16*). After puromycin selection, cells were split into five replicates of 2 × 10^7^ cells. Two replicates were cultured with the addition of DMSO, and two replicates were cultured in the presence of 3 μM olaparib for 14 days. One replicate was immediately collected as an input sample. Following 14 days of treatment, cells were collected, and genomic DNA was extracted using the NucleoSpin Blood XL kit (Macherey-Nagel). The sgRNA cassette was amplified using Q5 polymerase (New England Biolabs) as previously described (primers are shown in table S3) (*16*). Amplified DNA was sequenced using Illumina sequencing technology.

### CRISPR-screen analysis

Fifty–base pair (bp) single reads were trimmed using cutadapt (version 1.16) by removing the adapter sequence (-g GGACGAAACACCG) and setting the read length to 20 bp (-l 20). Trimmed reads were separately aligned to Human GeCKOv2 Library A or B using bowtie2 (version 2.3.4.1) with parameter --norc. Aligned reads were filtered by samtools (version 1.7) using the parameter -q 2. Reads were subsampled to 5 million reads with replacement and counted for each sgRNA, annotated with gene information, and libraries A and B from the same sample were merged into a table. Statistical analysis was performed using DrugZ (version1.1.0.2) (*18*). DrugZ outputs were visualized using R. Gene Ontology analysis was carried out using topGO (version 2.36.0) on the annotation org.Hs.eg.db (version 3.8.2) and GO.db (version 3.8.2) R packages. Cell fitness was determined by calculating the log_2_ fold change between DMSO and plasmid library sgRNA read counts (table S1).

### Live-cell imaging

U2OS WT or U2OS *ALC1^KO^* cells were seeded into eight-well Lab-Tek II chambered cover glass (Thermo Fisher Scientific) and transfected 48 hours before imaging. Cells were sensitized by aspirating growth medium from the Lab-Tek and replacing it with fresh medium containing Hoechst 33342 (0.15 μg/ml) for 1 hour at 37°C. Immediately before imaging, growth medium was replaced with CO_2_-independent imaging medium [phenol red–free Leibovitz’s L-15 medium (Life Technologies) supplemented with 20% FBS, 2 mM glutamine, penicillin (100 μg/ml), and streptomycin (100 U/ml)]. For PARP inhibition, cells were treated with olaparib (Euromedex) for 30 min before imaging. Hypotonic treatment was done as previously described (*40*).

Live-cell imaging experiments were completed on a Ti-E inverted microscope from Nikon equipped with a CSU-X1 spinning-disk head from Yokogawa, a Plan-Apochromat 60×/1.4–numerical aperture (NA) oil-immersion objective lens, and a sCMOS ORCA Flash 4.0 camera. The fluorescence of EGFP/PAGFP and mCherry/PTagRFP was excited with lasers at 490 and 561 nm, respectively. For fluorescence detection, we used band-pass filters adapted to the fluorophore emission spectra. Laser microirradiation and local photoactivation at 405 nm was performed along a 16-μm line through the nucleus using a single-point scanning head (iLas2 from Roper Scientific) coupled to the epifluorescence backboard of the microscope. To ensure reproducibility, laser power at 405 nm was measured at the beginning of each experiment and set to 125 μW at the sample level. Cells were maintained at 37°C with a heating chamber. Protein recruitment was quantified using a custom-made MATLAB (MathWorks) routine.

For FRAP experiments, we bleached using a 490-nm laser, a 20 × 20 pixel square located within the area previously irradiated at 405 nm to induce DNA damage. Images were collected at a frequency of 4 frames/s. After background subtraction, the fluorescence recovery kinetics were obtained by dividing the signal within the bleached area to the one measured in the unbleached part of the damaged region. FRAP curves were fitted with the following equation: *I*(*t*) = (1 − *FI*) × (1 − exp(−*t*/τ)), where τ is the characteristic recovery time of the fast population and FI is the immobile fraction.

### Analysis of PARP1 turnover at DNA lesions

Photoconversion of Dendra2-PARP1 proteins accumulating at DNA lesions was performed on a Zeiss LSM880 confocal setup equipped with a Plan-Apochromat 63×/1.2-NA water-immersion objective. DNA damage was induced by irradiating a 4 μm × 4 μm square of the cell nucleus with a pulsed infrared laser (Mai Tai, Spectra-Physics). Conversion of the green-to-red photoswitchable Dendra2 was achieved by illuminating at 405 nm the area previously irradiated with the infrared laser. The green and red forms of the Dendra2 were excited at 488 and 561 nm, respectively, and their emissions were detected at 500 to 560 nm and 590 to 700 nm, respectively. Cells were maintained at 37°C with a heating chamber.

### PAR3 hybrid assay

The PAR3H assay has been previously described (*39*). Briefly, U2OS-2B2 cells containing the LacO array were transfected with YFP-macrodomain of macroH2A1.1, LacI-GFP trap, and mCherry-PARP1 or mCherry-PARP1 E988K. Cells sensitized with Hoechst were irradiated with 405-nm light to induce DNA damage as described above. If PARP1 is PARylated, it will diffuse through the nucleus and interact with the macrodomain tethered at the LacO array and show an increase of mCherry intensity. Images were taken in a time lapse, and the intensity of mCherry at the LacO array was quantified before and 60 s after damage. The average intensity at the LacO array was normalized to the average intensity of the nucleus and corrected for background signal.

### Immunofluorescence

Cells were washed once with phosphate-buffered saline (PBS) before they were fixed with 3% paraformaldehyde (PFA) for 10 min. Cells were then permeabilized with 0.5% Triton X-100 in PBS for 10 min, washed three times with PBS, and incubated in blocking buffer (3% bovine serum albumin and 0.1% Triton X-100 in PBS) for 1 hour at room temperature (RT). Samples were incubated in primary antibody diluted in blocking buffer overnight at 4°C. Cells were washed three times with 0.1% Triton X-100 in PBS before incubation with fluorescently tagged secondary antibody (table S5) diluted in blocking buffer at RT for 1 hour in the dark. Cells were washed twice with 0.1% Triton X-100 in PBS and counterstained with Hoechst (1 μg/ml in PBS) for 10 min. Z-stacks of images were acquired on an LSM800 confocal setup with a Plan-Apochromat 20×/0.8 or a Plan-Apochromat 63×/1.4 oil objective and controlled by ZEN 2.3 software. Fluorescence excitation was performed using diode lasers at 405, 488, and 561 nm. Images were analyzed after generating the maximum intensity projections of the z-stacks. For Rad51 foci formation, cells were treated with olaparib (10 μM) for 48 hours before fixation and staining. The percentage of cells with >5 cells per nuclei was quantified. An average of 100 cells per condition were counted.

### X-ray and micropore irradiation assays

Cells seeded on coverslips were treated with 1 μM olaparib (Selleckchem) for 1 hour or were left untreated before irradiation. X-ray irradiation was performed at 90 kV and 150 mA with a dose of 2 Gy using a Trakis XR-11 x-ray machine. Cells were fixed with 3% PFA for 10 min at different time points after irradiation before immunofluorescence. γH2AX foci were counted in at least 40 cells per condition.

The micropore irradiation assay is based on a previously described protocol (*41*). Cells were labeled with 50 μM 5-bromo-2′-deoxyuridine (Sigma-Aldrich) for 24 hours followed by 1-hour treatment with 1 μM olaparib (Selleckchem) or were left untreated before UV irradiation. For UV irradiation, the medium was removed, and the cells were covered with polycarbonate membranes with a 5-μm pore size (Millipore) and exposed to 100 J/m^2^ of UV-C light. After UV irradiation, fresh cell medium matching the previous treatment was added to the cells, and the polycarbonate membrane was removed. Cells were washed for 3 min with preextraction buffer [10 mM tris-HCl, 2.5 mM MgCl_2_, 0.5% NP-40, and 100× protease inhibitor cocktail (Roche)] at 30 min (PARP1 analysis) or at 2 hours (pRPA, Ku70, Mre11, and RAD51 analysis) after irradiation before immunofluorescence processing. The percentage of the colocalization of pRPA, Ku70, Mre11, RAD51, or PARP1 with γH2AX foci was counted in at least 100 cells per condition.

### HR and NHEJ reporter assays

HeLa pGC or HeLa pEJ cells were transfected with siCtrl, siBRCA1, siKu70, or siALC1 siRNAs by DharmaFECT or mCherry-ALC1 plasmid construct by Xfect (Takara). Twenty-four hours after transfection, cells were transfected a second time with I-SceI plasmid constructs using Xfect (Takara) according to the manufacturer’s instructions. Forty-eight hours after I-SceI transfection, cells were fixed with 3% PFA for 3 min and stained with Hoechst. Z-stacks of images were acquired on an LSM800 confocal setup with a Plan-Apochromat 20×/0.8 or a Plan-Apochromat 63×/1.4 oil objective and controlled by ZEN 2.3 software. Fluorescence excitation was performed using diode lasers at 405, 488, and 561 nm. The raw images were analyzed in CellProfiler (*58*) after generating the maximum intensity projections of the z-stacks in Fiji (ImageJ) (*59*). In CellProfiler, the Hoechst-stained DNA was used to segment the nuclei. GFP-positive cells representing HR or NHEJ events and/or mCherry-positive cells representing ALC1 expression were counted and normalized to the total cell number. At least 5000 cells were analyzed per condition.

### Clonogenic survival assay

U2OS WT, *ALC1^KO^*, *PARP1^KO^*, and *ALC1^KO^ PARP1^KO^* double knockout cells were transfected with siRNA 48 hours before DNA damage and seeded in defined numbers 24 hours after transfection. Cells were treated with olaparib, veliparib, or niraparib (Selleckchem; 30, 300, and 3000 nM) for 24 hours or bleomycin (Sigma-Aldrich; 5, 10, and 50 μM), etoposide (Sigma-Aldrich; 5, 10, and 25 μM), or H_2_O_2_ (Molar; 5, 30, and 50 μM) for 1 hour. For combined olaparib and MMS treatments, cells were initially treated with olaparib (Selleckchem; 30, 300, and 3000 nM) for 24 hours before MMS (Sigma-Aldrich; 0.0003, 0.006, and 0.001%) was added to the cells for 1 hour. Cells were washed and incubated for 14 days. The obtained colonies were fixed with methanol:acetic acid (3:1) and stained with crystal violet (Sigma-Aldrich). The fraction of the surviving cells were calculated and normalized to nontreated conditions.

### Chromosome aberration analysis

U2OS WT, *ALC1^KO^*, and YFP-ALC1 cells were incubated with or without 1 μM olaparib for 24 hours. Cells were washed and then treated with colchicine (0.5 μg/ml; Sigma-Aldrich) for 6 hours to enrich for mitotic cells. For the preparation of chromosome spreads, cells were collected, resuspended in 75 mM KCl for 25 min at 37°C, centrifuged at 200*g* at 4°C for 10 min, and then fixed (3:1 methanol: acetic acid) three times. Chromosomes were spread onto coverslips, air-dried, and stained with acridine orange (Sigma-Aldrich, at 1:10,000). For each experiment, images of 40 chromosome spreads were captured on an LSM800 confocal setup, and the number of chromosome aberrations per chromosome spread was counted.

### Cell cycle analysis by flow cytometry

Cells were incubated with or without 1 μM olaparib for 24 hours. Cells were then collected and fixed with 70% EtOH for 30 min on ice. Cells were washed with PBS and treated with ribonuclease A solution (10 μg/ml; Sigma-Aldrich) for 20 min at RT. DNA content of the cells were stained with propidium iodide solution [0.1% Triton X-100 and propidium iodide (500 μg/ml); Sigma-Aldrich] for 10 min. G_0_-G_1_, S phase, and G_2_-M were differentiated by a flow cytometer (BD FACSCalibur), using BD CellQuest Pro version 6.0, ModFit LT software.

### γH2AX intensity assay

Cells were plated onto glass-bottom cell culture chambers (Greiner). Twenty-four hours later, the coverslips with different cell types were treated or not treated with different concentrations of olaparib (30, 300, 3000, and 30,000 nM) for 12 hours and analyzed by immunofluorescence. Nuclei were visualized by Hoechst staining. The raw images were analyzed in Fiji (ImageJ) (*59*) after generating the maximum intensity projections of z-stacks and analyzed in CellProfiler (*58*). The Hoechst-stained DNA was used to segment the nuclei, and the intensity of γH2AX signal is plotted normalized to the untreated controls in different cell types.

### EdU and IdU incorporation assay

Cells were labeled with 10 μM EdU (Baseclick, BCK-EdU555) for 30 min and washed with fresh culturing medium. Cells were then treated or not treated with olaparib (3 μM) for 1 hour and labeled with 200 μM IdU (Sigma-Aldrich) thymidine analog for 30 min. Cells were fixed with 3% PFA for 10 min and treated with 2.5 M HCl to denature the DNA. Cells were washed three times with PBS and immunostained with anti-IdU antibodies. EdU incorporation was visualized by a Click-iT kit (Baseclick) according to the manufacturer’s instructions. Nuclei were stained with Hoechst. The EdU and IdU foci related to the replication fork movement were imaged with an LSM800 confocal setup with a Plan-Apochromat 20×/0.8 or a Plan-Apochromat 63×/1.4 oil objective and controlled by ZEN 2.3 software. Fluorescence excitation was performed using diode lasers at 405, 488, and 561 nm.

### Visualization of the chromatin fraction of PARP1-mCherry in cells

HeLa-PARP1-mCherry cells were transfected with siCtrl, siBRCA1, or siALC1 siRNAs, and then 24 hours later, cells were plated onto glass-bottom cell culture chambers (Greiner). Forty-eight hours after the transfection, cells were incubated with or without 1 μM olaparib for 1 hour and then washed with pre-extraction buffer (10 mM tris-HCl, 2.5 mM MgCl_2_, 0.5% NP-40, and protease inhibitor cocktail 1:100) for 3 min and fixed with 3% PFA for 5 min. The nuclei were stained with Hoechst. At least 6000 cells per condition were captured using an LSM800 confocal setup with a Plan-Apochromat 20×/0.8 or a Plan-Apochromat 63×/1.4 oil objective and controlled by ZEN 2.3 software. Fluorescence excitation was performed using diode lasers at 405, 488, and 561 nm. The raw images were analyzed in CellProfiler (*58*) after generating the maximum intensity projections of the z-stacks in Fiji (ImageJ) (*59*). In CellProfiler, the Hoechst-stained DNA was used to segment the nuclei. The intensity of PARP1-mCherry signal is plotted in different cell types normalized to the untreated sample.

### Alkaline comet assay

U2OS WT or *ALC1^KO^* cells were incubated with 1 μM olaparib for 1 hour (fig. S3D) or for 24 hours (fig. S2E) or were left untreated before irradiation. X-ray irradiation was performed at 90 kV and 150 mA with a dose of 2 Gy using a Trakis XR-11 x-ray machine. Cells were collected 30 min after x-ray treatment and washed with 1× PBS. Cells were in diluted 1% low-gelling-temperature agarose (approximately 5000 cells/ml) and rapidly dropped onto 1% agarose-covered surface of precoated microscope slides. After agarose has gelled, samples were lysed in alkaline lysis solution [1.2 M NaCl, 100 mM Na_2_EDTA, 0.1% sodium lauryl sarcosinate, and 0.26 M NaOH (pH > 13)] overnight at 4°C in the dark. Samples were then washed with rinse solution [0.03 M NaOH and 2 mM Na_2_EDTA (pH ∼ 12.3)] for 20 min. Microscope slides were then submerged in an electrophoresis chamber containing fresh rinse solution, and electrophoresis was run for 25 min at a voltage of 0.6 V/cm (*60*). Comets were visualized by Hoechst staining. One hundred comet images from each slide were analyzed in Fiji (ImageJ) (*59*) after generating the maximum intensity projections of z-stacks. The tail moment lengths—the distance between the centers of the nucleus and the tail—were plotted.

### Chromatin fractionation

Cells were incubated with 1 μM olaparib for 1 hour or were left untreated before irradiation. X-ray irradiation was performed at 90 kV and 150 mA with a dose of 2 Gy using a Trakis XR-11 x-ray machine. Cells were collected 30 min after x-ray treatment and washed with 1× PBS. The Subcellular Protein Fractionation Kit (Thermo Fisher Scientific) was used for chromatin fractionation according to the manufacturer’s instructions. The input (whole cell extract, WCE) and chromatin fraction (chromatin fraction) protein extracts were prepared for SDS–polyacrylamide gel electrophoresis in 5× sample buffer (10% SDS, 300 mM Tris-HCl, 10 mM β-mercaptoethanol, 50% glycine, and 0.02% bromophenol blue). Separated proteins were transferred onto nitrocellulose membrane, blocked for 1 hour at RT in 5% low-fat milk in 0.1% tris-buffered saline, and incubated with primary antibodies overnight at 4°C. Membranes were then washed and incubated with horseradish peroxidase–conjugated secondary antibodies for 1 hour. Nitrocellulose membranes were developed with enhanced chemiluminesence using UviTec machine and Alliance Q9 Advanced software.

### Quantification and statistical analysis

All data were derived from at least *n* = 3 replicates, and for each experiment, at least 40 nuclei or chromosome spreads were analyzed. A minimum of 20 cells per condition were examined in live-cell imaging experiments. *P* values from live-cell imaging experiments (Fig. 4 and fig. S4) were calculated using an unpaired Student’s *t* test with Bonferroni correction (*P < 0.05, **P < 0.01, and ***P < 0.001). Statistical analysis was performed using R statistical software (version 3.6). Linear regression models or linear mixed effect models were fitted using lm (stats package) or lmer (lme4 package version 1.1-23 and lmerTest package version 3.1-2) functions, respectively. Dose-response curves were analyzed similarly as previously published (*61*). Specifically linear, quadratic, or cubic regression models were fitted using orthogonal polynomials of degree 1, 2, or 3, respectively (stats package poly function). The final model was chosen by the Akaike information criterion (bbmle package version 1.0.23.) and by diagnostics of the residuals (DHARMa package version 0.3.1). Plots were generated by ggplot2 package (version 3.3.1). Asterisks represent *P* values, which correspond to the significance of regression coefficients (*P < 0.05, **P < 0.01, and ***P < 0.001). For second- or third-degree polynomial models, the lowest *P* value is chosen from linear, quadratic, or cubic terms, as a significant difference in any of these coefficients indicates different curve characteristics. Model summaries are provided in table S2. Detailed analysis code is available on GitHub: (https://github.com/tschauer/Juhasz_etal_2020).

## SUPPLEMENTARY MATERIALS

Supplementary material for this article is available at http://advances.sciencemag.org/cgi/content/full/6/51/eabb8626/DC1

## Appendix

View/request a protocol for this paper from *Bio-protocol*.
